# Defining the Soluble and Extracellular Vesicle Protein Compartments of Plasma Using In-Depth Mass Spectrometry-Based Proteomics

**DOI:** 10.1021/acs.jproteome.4c00490

**Published:** 2024-08-14

**Authors:** Nidhi Sharma, Silvia Angori, AnnSofi Sandberg, Georgios Mermelekas, Janne Lehtiö, Oscar P. B. Wiklander, André Görgens, Samir El Andaloussi, Hanna Eriksson, Maria Pernemalm

**Affiliations:** † Department of Oncology-Pathology, Karolinska Institute, 171 77 Stockholm, Sweden; ‡ Theme Cancer, Skin Cancer Center, Karolinska University Hospital, 171 77 Solna, Sweden; § Biomolecular Medicine, Clinical Research Center, Department of Laboratory Medicine, Karolinska Institute, 171 76 Solna, Sweden; ∥ Institute for Transfusion Medicine, University Hospital Essen, University of Duisburg-Essen, 45141 Essen, Germany; ⊥ Science for Life Laboratory, Solna, Tomtebodavägen 23, 171 65 Solna, Sweden

**Keywords:** extracellular vesicles, plasma, proteomics, mass spectrometry, melanoma, lung adenocarcinoma

## Abstract

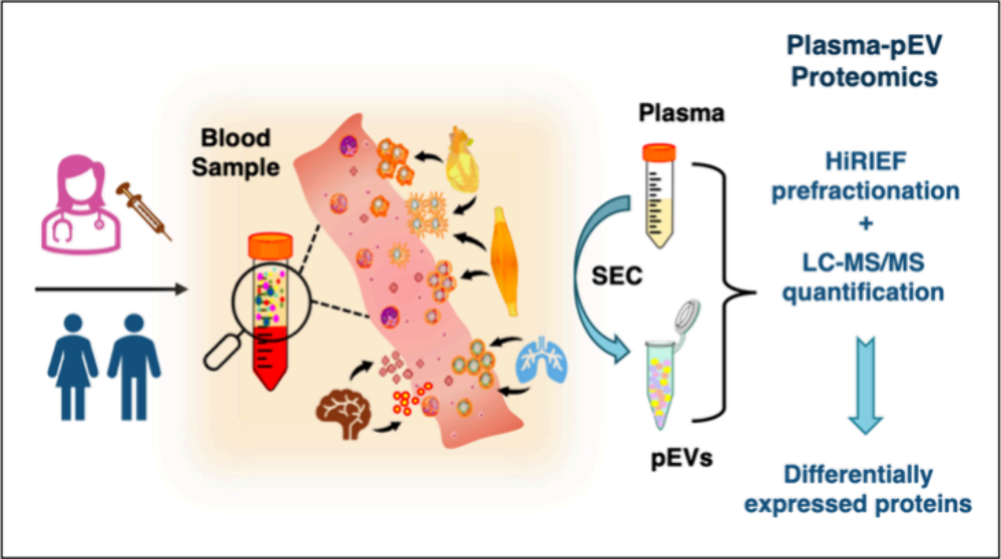

Plasma-derived extracellular vesicles (pEVs) are a potential source of diseased biomarker proteins. However, characterizing the pEV proteome is challenging due to its relatively low abundance and difficulties in enrichment. This study presents a streamlined workflow to identify EV proteins from cancer patient plasma using minimal sample input. Starting with 400 μL of plasma, we generated a comprehensive pEV proteome using size exclusion chromatography (SEC) combined with HiRIEF prefractionation-based mass spectrometry (MS). First, we compared the performance of HiRIEF and long gradient MS workflows using control pEVs, quantifying 2076 proteins with HiRIEF. In a proof-of-concept study, we applied SEC–HiRIEF–MS to a small cohort (12) of metastatic lung adenocarcinoma (LUAD) and malignant melanoma (MM) patients. We also analyzed plasma samples from the same patients to study the relationship between plasma and pEV proteomes. We identified and quantified 1583 proteins in cancer pEVs and 1468 proteins in plasma across all samples. While there was substantial overlap, the pEV proteome included several unique EV markers and cancer-related proteins. Differential analysis revealed 30 DEPs in LUAD vs the MM group, highlighting the potential of pEVs as biomarkers. This work demonstrates the utility of a prefractionation-based MS for comprehensive pEV proteomics and EV biomarker discovery. Data are available via ProteomeXchange with the identifiers PXD039338 and PXD038528.

## Introduction

Extracellular vesicles (EVs) are cell-derived heterogeneous populations of nano- to microsized membrane-limited particles released into the extracellular environment. EVs carry producer cell-type specific proteins that define their secretion, signaling targets and fate, playing essential roles in both normal physiology and pathology by transporting unique molecular cargo to target cells.^1^ EVs are classified into exosomes (30–200 nm), microvesicles (100–1000 nm), and apoptotic bodies (>1000 nm), based on size and biogenesis, and are described by their cell of origin, cell state, and release method.^2,3^ Clinical research on EVs as cancer biomarkers has increased significantly during the past decade,^4^ revealing their potential to define various human cancers like melanoma, lung cancer, breast cancer, and glioblastoma.^5−7^ EVs are actively involved in regulating tumor cell proliferation, epithelial-mesenchymal transition (EMT), immune evasion, and premetastatic niche formation.^8^ However, the majority of EV research has been conducted on tissue- and cell-derived EVs, and it cannot be directly translated to humans, highlighting the need for more research on plasma-derived EVs (pEVs).

Although pEVs are a valuable source of clinically useful information, they are often overlooked in plasma biomarker discovery studies due to the relatively low abundance of pEV proteins compared to the total plasma protein content, necessitating specific EV enrichment strategies.^9^ The process of EV isolation and subsequent exploration of the pEV proteome for biomarker discovery, is largely limited by the coenrichment of highly abundant plasma proteins like lipoproteins, biological factors including large inter and intrapatient variability, and the sensitivity of applied proteomics methods. To date, only a handful of pioneer studies have analyzed human pEVs with modest EV proteome coverage, utilizing mass-spectrometry (MS)-based workflows.^10^ The number of proteins identified in these studies were far lower than expected, compared to the number of proteins detected from cell-derived EV samples, where thousands of proteins are commonly identified.^7,11,12^ We, along with others, have previously shown that the choice of EV source (i.e., plasma or serum), amount of starting material, and method of isolation greatly influence the enrichment of EVs from the non-EV proteins in human plasma, affecting the analytical depth and EV proteome coverage in downstream MS analysis.^11,13^

Given these challenges in isolating and analyzing pEVs, selecting the appropriate enrichment method is crucial for successful proteome profiling. Currently, well-known EV isolation methods include ultracentrifugation, immuno- or affinity-based enrichment, and size exclusion chromatography (SEC) for the enrichment of total EV populations or specific EV subtypes. Recently developed EVtrap, a functionalized magnetic-bead-based method, is robust, efficient, and scalable and requires a small sample volume. However, as a novel method, EVtrap currently has limited widespread validation and may require further optimization. In contrast, SEC separates analytes based on size, allowing for the relative enrichment of EVs compared to small lipoproteins and other soluble proteins present in the plasma.^14^ Recent findings have shown that SEC of platelet-poor plasma outperforms other EV isolation methods in the detection of EV markers and is compatible with global proteomics analysis of pEVs.^11,13^ Therefore, we used SEC for the enrichment of EVs from the plasma.

To further enhance the depth of proteome profiling, a highly sensitive liquid-chromatography tandem mass spectrometry (LC–MS/MS) proteomics workflow must complement the EV isolation method. Recent advances in LC–MS/MS instrumentation and prefractionation-based MS methods have been widely applied to proteome profiling in complex samples. Prefractionation, which involves adding a fractionation step before the MS analysis, increases proteome coverage. Common strategies include sodium dodecyl-sulfate polyacrylamide gel electrophoresis (SDS-PAGE), strong-cation-exchange (SCX) chromatography, and high/low pH reversed-phase chromatography.^15^ High Resolution Iso-Electric Focusing (HiRIEF) is another effective prefractionation method that separates peptides based on their isoelectric points (pI), reducing complexity and improving the identification of more peptides and proteins, especially low-abundance proteins present in complex mixtures. Whereas, the basic pH reverse phase and ion exchange separation methods separate peptides based on hydrophobicity and charge, respectively; both methods can result in overlapping peptide peaks and may not achieve the same level of resolution as HiRIEF. Therefore, HiRIEF provides more consistent peptide elution profiles compared to basic pH reverse phase or ion exchange methods, which is crucial for accurate quantification in MS. Our study explores the utilization of HiRIEF–MS for achieving high proteome coverage in pEVs, due to its advantages over other methods and its demonstrated potential in clinical proteomics analysis. HiRIEF–MS, an unbiased global method for discovery proteomics,^16^ has previously been proven to be compatible with clinical proteomics analysis of patient cohorts, with its high proteome coverage and ability to detect candidate disease-specific markers in both plasma and tissue-based studies.^17,18^

**Figure 1:**
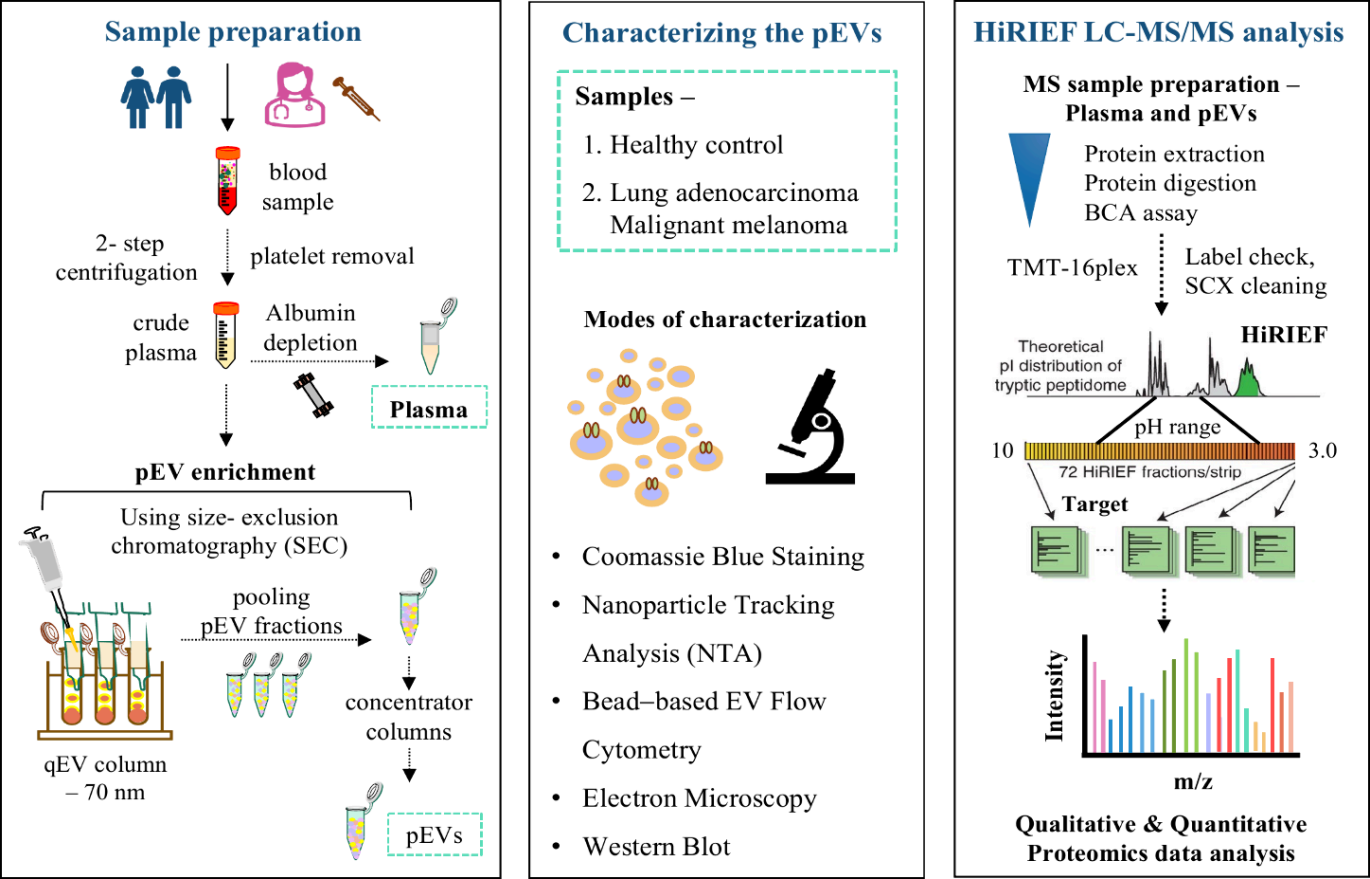
Study design and workflow for in-depth proteome profiling of plasma-derived EVs (pEVs) using the SEC–HiRIEF–MS workflow. The EVs were separated from plasma using size exclusion chromatography (SEC) and characterized using Coomassie blue staining, nanoparticle tracking analysis (NTA), bead-based flow cytometry analysis, transmission electron microscopy (TEM) and Western blot. We first evaluated the performance of HiRIEF– and conventional long-gradient–MS approach for comprehensive proteome profiling of pEVs using healthy control plasma. Subsequently, we implemented the SEC–HiRIEF–MS workflow in cancer patients for deep comparative proteome analysis of both plasma and the separated cancer pEVs in parallel.

The SEC coupled HiRIEF–MS provides an effective methodology for detailed and accurate proteomic profiling of pEVs, facilitating the identification of clinically relevant protein changes and advancing clinical proteomics. In the present study, we aimed at achieving in-depth quantitative proteome profiling of pEVs from only 400 μL of plasma using SEC–HiRIEF–MS analysis as a measure to improve proteome coverage in pEV studies (Figure 1). For this purpose, we first employed the SEC–HiRIEF–MS workflow for pEV proteomics using plasma from a healthy control. We then compared the performance of the HiRIEF– and conventional long-gradient (LG)–MS methods for pEV proteomics. Second, as a proof-of-concept, we evaluated the applicability of SEC–HiRIEF–MS in cancer biomarker detection for plasma and pEVs, using plasma from metastatic lung adenocarcinoma (LUAD) and metastatic malignant melanoma (MM) patients. Our selection of cancer cohort was motivated by recently published studies that highlight the role of EVs in shaping tumor–stroma interactions, which facilitate melanoma and lung cancer metastasis. These studies also demonstrate the potential use of pEVs as biomarkers to monitor tumor progression and treatment monitoring.^19,20^ We have systematically compared the depleted plasma and pEVs side by side to fundamentally understand the relationship between the total plasma and pEV proteomes. By applying SEC–HiRIEF–MS to pEVs of cancer patients in parallel with quantitative proteome profiling of plasma, we demonstrate that pEVs exhibit a distinct protein signature in cancer patients compared to that in the plasma, albeit in a small test cohort (Figure 1). Therefore, we suggest that expanding the application of a prefractionation-based MS approach to pEV proteomics could significantly enhance protein identification and quantification, facilitate novel EV protein biomarker discovery, and extend its use to other clinical studies focused on biomarker discovery.

## Experimental Procedures

### Study Design

The study was approved by the Regional Ethical Review Board in Stockholm, Sweden and by the Swedish Ethical Review Authority and is in accordance with the Declaration of Helsinki. For MS workflow optimization, a plasma sample from one healthy volunteer was used as a control. Here, we used a plasma sample from one healthy volunteer as a control for optimizing MS workflow for pEVs and making HiRIEF and LG method comparisons. In the cancer cohort, samples were taken at diagnoses before any specific treatment for cancer was started. The samples are balanced on age, gender, and late stage patients of same histology. Plasma from six patients diagnosed with stage IV LUAD and six with stage IV MM was analyzed (Figure 4a). In addition, we used experimental and technical triplicates for the S6 and S12 patients; therefore, a total of 16 pEV samples and 16 corresponding plasma samples were analyzed in parallel. Due to limited plasma sample availability, we could perform replicates only for two of the samples. The samples were selected randomly for triplicates.

### Plasma Sample Collection and Preprocessing

All patients and healthy controls included in this study provided written informed consent to participate in the study. The samples were not taken from fasting donors. To avoid coagulation and minimize protein degradation, the whole blood plasma samples were collected in EDTA tubes (BD Vacutainer K2E 7.2 mg, BD Diagnostics) and stored at 4 °C until preparation. We used the standard two-step centrifugation protocol as per the ISTH Guidelines, for separation of plasma from the cellular component of blood to reduce the number of platelets in plasma.^21^ Here, the EDTA tubes were first centrifuged at 1500*g* at 4 °C for 10 min and the supernatant was transferred to a new tube for centrifugation at 3000*g* at 4 °C for 10 min. The resulting supernatant is commonly referred to as the “platelet-poor-plasma”;^22^ however, it would still contain some platelet residues as discussed in the reported findings. The collected plasma was stored at 80 °C until analysis.

### High Abundant Plasma Protein Depletion

We used 10 μL of plasma from each patient sample. The highly abundant plasma proteins were depleted using the Pierce Top14 Abundant Protein Depletion Resin Kit. For the TMT analyses, the depleted plasma flow-through was concentrated on a 5 kDa molecular weight cutoff filter, followed by a buffer exchange to 50 mM HEPES pH 7.6.

### SEC-Based pEV Enrichment

For EV isolation, 400 μL of plasma was preprocessed by centrifugation at 1500*g* for 10 min (4 °C), and at 10,000*g* for 10 min (4 °C) to remove any cells and large particles. The centrifuged plasma was used as the input for SEC. EVs were isolated using size exclusion chromatography (SEC)-based qEV/70 nm columns with an optimum particle recovery size ranging between 70 and 1000 nm (IZON Science Ltd.) as per the manufacturer’s protocol. Dulbecco’s phosphate-bufered saline (DPBS) (Gibco, 14190250) was used as the solvent for SEC fractionation. Prior to SEC fractionation, the column was washed with at least 20 mL of filtered and degassed DPBS. A constant solvent flow was maintained to avoid drying of the columns. The first 3 mL was discarded as the void volume, and the next three fractions, each measuring 0.5 mL, were collected and pooled to form the EV enriched fraction (F1). Additionally, the four 1.5 mL fractions (F2–F5) that were eluted right after the pEVs were also saved for quality control analysis. The pEVs were concentrated using polymeric strong cation spin columns (Pall Microsep, PES, 10 kDa molecular weight cut off filter). Each sample-loaded column was centrifuged at 1957*g* for 10–15 min.

### EV Characterization

#### Coomassie Brilliant Blue (CBB) Gel Staining

The samples were loaded onto a 4–12% gel (ThermoFisher Scientific NuPAGE protein gel) and run for 80 min at 160 V. We have used two gel lanes for each patient: first lane, pEVs and second lane, other plasma fractions (pooled) eluted right after the EVs (F2–F5). As a control, a 3–198 kDa SeeBlue Plus2 prestained protein ladder was used. The CBB staining solution recipe available from the Cold Spring Harbor Protocols and the iBright CL750 Imaging System were used for protein band staining and visualization.

#### Nanoparticles Tracking Analysis (NTA)

The particle concentration and size distribution analysis was performed for the F1 (concentrated pEVs) and F2 fractions of the sample, and measured using an NS500 nanoparticle analyzer (NanoSight, Malvern, Worchestershire, UK) as described.^23^ To summarize, all samples were diluted in PBS at a ratio of 1:10 and 1:50 to achieve a particle count between 1 × 10^8^ and 1 × 10^9^ per mL. The camera level was set to ten, and the camera focus was adjusted so that the particles appeared as sharp dots. Five 30-s videos were recorded for each sample using the script control function, with a sample advance and a 5-s delay between each recording. Except for the detection threshold, which was fixed at 2, all postacquisition settings were automated.

#### Bead-Based EV Flow Cytometry

Plasma samples were subjected to multiplex bead-based flow cytometry analysis (MACSPlex Exosome Kit, human, Miltenyi Biotec) to quantify EV surface marker expression levels of pEVs, as previously described.^24^ In brief, pEVs prepared by SEC (F1 fraction) were stored at −80 °C in PBS-HAT until use, and amounts corresponding to equal initial plasma amounts before processing (total volume 60 μL) were loaded in wells of a prewet and drained MACSPlex (96 well, 0.22 m filter) plate, followed by 8 μL/well of MACSPlex Exosome Capture Beads. Filter plates were incubated at 450 rpm at room temperature (RT) overnight. The beads were washed with 200 μL of buffer, and the liquid was removed with a vacuum manifold (Sigma-Aldrich, Supelco PlatePrep; −100 mbar). A mixture of APC-conjugated anti-CD9, anti-CD63, and anti-CD81 detection antibodies (supplied in the kit; 5 μL each) was added to each well for counterstaining of EVs bound by capture beads with detection antibodies, and the plate was incubated for 1 h at RT. The samples were then washed twice and resuspended in MACSPlex buffer before being transferred to V-bottom 96 well microtiter plates (ThermoFisher Scientific). A MACSQuant Analyzer 10 flow cytometer was used for data acquisition (Miltenyi Biotec). Flow cytometric data was analyzed and visualized using the FlowJo (v10.8.1) and MPAPass (v1.01) softwares. To determine the normalized staining intensity, the median fluorescence intensities obtained from pEV samples for all 39 capture bead subsets (EVs + capture beads + antibodies) were normalized by calculating respective fold change values for each capture bead subset over control setup beads and log10 data set scaling as described before.^25^

#### Transmission Electron Microscopy (TEM)

For negative stain TEM, 3 μL of the sample was applied on glow discharged carbon coated and Formvar stabilized 400 mesh copper grids (Ted Pella) and incubated for approximately 30 s. The excess of the sample was blotted off, and the grid was washed with Milli-Q water prior to negative staining using 2% uranyl acetate (EMS). TEM imaging was done using a Hitachi HT7700 transmission electron microscope operated at 100 kV equipped with a 2kx2k Veleta CCD camera (Olympus Soft Imaging System).

#### Western Blot

Prior to determination of the protein abundance, plasma samples were diluted 1:5 in 1× PBS. A BCA assay was used to determine the protein concentration of plasma and pEV samples (Pierce protein assay kit, Thermo Fisher, #23225). Equal protein concentrations (15 μg/sample) of plasma and pEVs were loaded on each lane. Except for sample MM–4, where we applied relative concentration and loaded 7.66 μg/sample for both plasma and pEV lane, due to the low pEV protein concentration. Western blot analysis was performed as described below. Each sample was mixed with a loading buffer (Laemmli buffer 4×; #1610747) and DTT (1:10; Cell Signaling, #7016L) and then heated to 75 °C for 10 min. All Western blot reagents and equipment were purchased from Bio-Rad, unless specified otherwise. Proteins were separated by denaturation on an 10% Mini-Protean TGX Precast Gel (#456-1033) at 200 V for 30 min, and then transferred onto a nitrocellulose membrane (#20221017) using a Trans-Blot Turbo Transfer system. The membranes were subsequently blocked in 1× TBS with 0.1% Tween (TBST) containing 5% (w/v) nonfat dry milk for 1 h at RT. The membranes were incubated with primary antibodies in blocking solution at 4 °C overnight. The following primary antibodies (1:500) were used: ALIX (Abcam, Ab186728), FLOT1 (Abcam, Ab133497), and APOA1 (Abcam, Ab52945). Following 3 × 10 min washes in TBST, membranes were incubated in HRP tagged antirabbit or antimouse IgG antibody (1:2000, Cell signaling, #7074 and #7076) in 5% Milk-TBST for 1 h at RT. The blots were then washed again for 3× 10 min. Proteins were visualized by adding Pierce ECL Western Blotting Substrate (#32016) with ChemiDoc detection system. After detection, the membranes were stained with Ponceau S solution (ThermoFisher, A40000279) and visualized with a ChemiDoc system.

### Plasma Protein Extraction and Digestion

The depleted plasma was denatured at 95 °C for 5 min, followed by reduction with dithiothreitol (DTT; final concentrations of 1 mM) at 95 °C for 30 min and alkylation with chloroacetamide (CAA; final concentrations of 4 mM) at RT for 20 min. After protein concentration measurement, 25 μg of protein per sample was taken for in-solution digestion with Lys-C and trypsin (1:50 ratio, 37 °C overnight). All of the peptides were collected and stored at −20 °C before TMT labeling.

### pEV Protein Extraction and Digestion

The pEV samples were lysed in a 2% SDS lysis buffer and prepared for MS analysis in accordance with a modified version of the SP3 protein clean up and digestion protocol.^26^ The protein samples were reduced and alkylated in the same manner as plasma. A Sera-Mag SP3 bead mix (10 μg/μL, 20 μL) was added to each protein sample, followed by the addition of 100% acetonitrile (ACN), for a final concentration of 70%. The bead–protein mixture was incubated under rotation at RT for 18 min. The mixture was then placed on a magnetic rack and the supernatant was discarded, after which two washes with 70% ethanol and one with 100% ACN was performed. The beads–protein mixture was first incubated overnight at 37 °C with 100 μL of a Lys-C buffer (0.5 M Urea, 50 mM HEPES [pH 7.6]; 1:50 enzyme-to-protein ratio), followed by overnight incubation with 100 μL of trypsin (50 mM HEPES [pH 7.6]; 1:50 enzyme-to-protein ratio). After digestion, the mixture was moved on the magnetic rack and the peptide mixture was eluted and collected into fresh tubes. For SP3 peptide cleanup, 20 μL of a SP3 bead mixture was added to each sample and 100% ACN was added to achieve a final concentration of >95%. Pipette-mixed samples were incubated at RT for 18 min under rotation. The sample tubes were placed on a magnetic rack, and the supernatant was removed. The beads were washed twice with 200 μL of 100% ACN before being removed from the magnetic rack. The beads were reconstituted in 100 μL of a phase A (3% ACN, 0.1% FA) buffer solution, and incubated at RT for 10 min. After the beads were placed on a magnetic rack, the supernatant with peptides was carefully collected and transferred to an MS-vial. The peptide concentration was determined using the Bio-Rad DC Assay and 50 μg of peptides per sample was collected for TMT labeling.

### Mass Spectrometry Analyses

#### LG-based LC–MS

An aliquot of 10 μg of peptides was suspended in LC mobile phase A. The LC–MS analysis was performed using a Dionex UltiMate 3000 RSLCnano System coupled to a High Field Q-Exactive mass spectrometer (Thermo Scientific). Here, 1 μg of sample was injected on the LC–MS system. Samples were trapped on a C18 guard desalting column (Acclaim PepMap 100, 75 μm × 2 cm, nanoViper, C18, 5 μm, 100 Å) and separated on a 50 cm-long C18 column (Easy spray PepMap RSLC, C18, 2 μm, 100 Å, 75 μm × 50 cm). The nano capillary solvent A contained 95% water, 5% DMSO, 0.1% FA; solvent B contained 5% DMSO, 95% ACN, 0.1% FA. At a constant flow of 0.25 μL/min^–1^, the curved gradient went from 6% B up to 43% B in 180 min, followed by a steep increase to 100% B in 5 min.

FTMS master scans with 60,000 resolution (and mass range 300–1500 *m*/*z*) were followed by data-dependent acquisition MS/MS (30,000 resolution) on the top 5 ions using higher energy collision dissociation at 30% normalized collision energy. Precursors were isolated with a 2 *m*/*z* window. Automatic gain control (AGC) targets were 1e6 for MS1 and 1e5 for MS2. Maximum injection times were 100 ms for MS1 and MS2. The entire duty cycle lasted ∼2.5 s. Dynamic exclusion was used with 60 s duration. Precursors with an unassigned charge state or charge state 1 were excluded. An underfill ratio of 1% was used.

#### Tandem Mass Tags (TMT)-Labeling

The peptides were TMT-labeled in accordance with the manufacturer’s instructions (Thermo Scientific). We tried to reduce TMT batch-induced technical bias by running all pEV samples together in a TMT16-plex, and similarly for plasma samples. Therefore, separate TMT16-plex sets were used for plasma and pEVs samples. The peptides were pH adjusted using a triethylammonium bicarbonate (TEAB) buffer, pH 8.5, and labeled with isobaric TMT16-plex labels for each set. The labeling efficiency was evaluated by LC–MS/MS on pooled samples using 30 min gradients to ensure >95% labeling of peptides before pooling. Following label check, all samples were pooled together and desalted using strong cation exchange (SCX) cleanup with solid phase extraction columns before taken for LC–MS/MS analysis. The eluted peptides were dried (SpeedVac) and stored at −20 °C for the next step.

#### HiRIEF LC–MS

The HiRIEF method was used as described previously.^16^ We used peptide isoelectric focusing by an immobilized pH gradient (IEF-IPG) in the pI range 3–10. The TMT-labeled peptide pool was dissolved in 250 μL of a rehydration solution containing 8 M urea, bromophenol blue, and 1% IPG Pharmalyte with a pH range of 3–10 (GE Healthcare). The peptide mixture was loaded onto an IPG gel strip (24 cm; linear gradient) and incubated overnight for adsorption by swelling. The gel strip was first focused, and then the peptides were extracted from the gel into 72 contiguous fractions (Milli-Q water/35% ACN/35% ACN, 0.1% FA) collected in a 96-well plate (V-bottom, Greiner 651201) using our in-house IPG extractor robot (GE Healthcare Biosciences AB). The fractions obtained were freeze-dried and kept at −20 °C for LC–MS analysis. For the MS run, each dried fraction was dissolved in 20 μL phase A and 10 μL was finally injected using an online 3000 RSLC nano system coupled to a Thermo Scientific Q Exactive-HF. The MS run was performed using an online 3000 RSLC nano system coupled to a Thermo Scientific Q Exactive-HF. Each nonlabeled and TMT-labeled HiRIEF plate run for all fractions was completed in 67 h. The 72 fractions from the HiRIEF prefractionation were concatenated to 40, and the analysis was performed using a dynamic gradient scheme (Datasheet S2).

#### LC–MS/MS Data Search and Quantification

The database search was performed using our in house proteomics pipeline (v2.9) built using Nextflow (v20.01.0), MSGF+ (v2020.03.14), Dinosaur (v1.2.0), and Percolator (v3.04.0) tools in the Galaxy platform to match MS spectra to the human Ensembl version 103 protein database. MSGF+ settings included precursor mass tolerance of 10 ppm, fully tryptic peptides, maximum peptide length of 50 amino acids, and a maximum charge of 6. Fixed modifications were TMT-16plex on lysines and peptide N-termini and carbamidomethylation on cysteine residues, and a variable modification was used for oxidation on methionine residues. Quantification of TMT-16plex reporter ions was done using OpenMS project’s Isobaric Analyzer (v2.5.0). PSMs found at 1% FDR (false discovery rate) were used to infer gene and protein identities.

### EV Protein Reference Lists

We created an EV protein reference list of 107 most observed EV proteins that includes the top 100 EV proteins listed in the ExoCarta database and 13 novel pan-EV marker proteins for EVs in humans, as reported by Hoshino et al.^5^ (Datasheet S1). We have also used the human plasma and pEV proteome provided by the PeptideAtlas for comparison.^27^

### Statistical Analysis

Proteins with quantitative values across all of the samples were included in MS analysis. The output data was gene-centric, and a gene table with unique ensemble gene ID’s was used for computational analysis. In SEC–HiRIEF–MS, the TMT data are median normalized and MS output data includes the protein quantification values in form of log2-normalized TMT ratios. We first observed normal data distribution using histogram plots and later confirmed data normality by applying the Shapiro–Wilk test; approximately 80% of the proteins exhibited a normal distribution across different samples in both the plasma and pEV proteome data sets. The DEPs in the plasma and EV data sets were also identified using the *t* test. The changes in protein levels were quantified with a log2-FC value. All the quantitative proteome analyses and data visualization was performed using R V.4.0.4 in the RStudio environment.^28^ The Database for Annotation, Visualization, and Integrated Discovery (DAVID) tool was used for gene Ontology (GO) terms association with plasma and EV proteomes, using the human genome as a background. The threshold was set to modified Fisher exact P-value ≤0.05 from the Benjamini method.

## Results

### Characterization of Plasma-Derived Extracellular Vesicles

**Figure 2:**
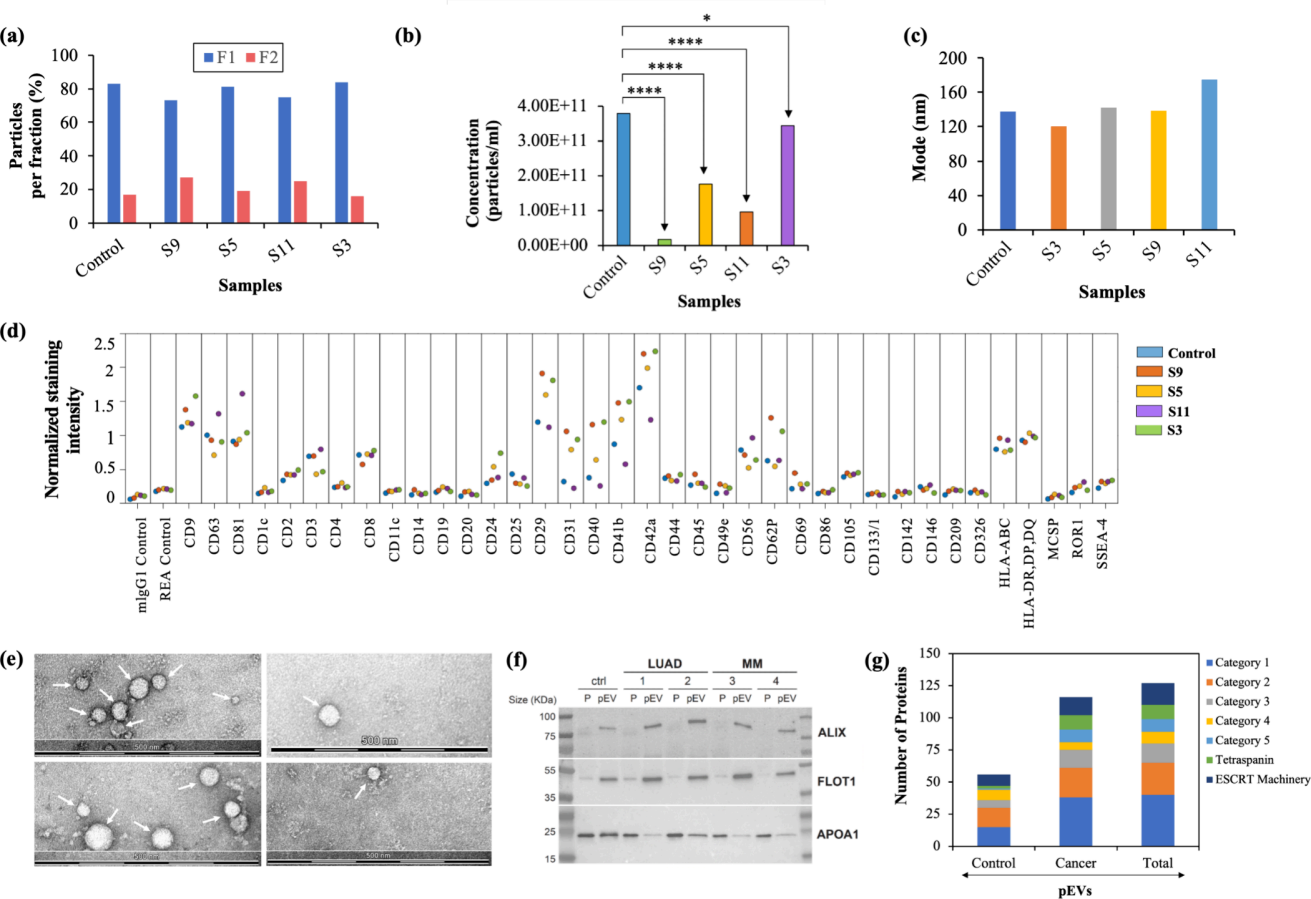
Characterization of plasma-derived extracellular vesicles (pEVs). The pEVs were enriched from healthy control and patients with metastatic MM and LUAD using the SEC columns. (a–c) Size distribution and concentration of isolated particles in control and cancer pEVs (MM—S3, S5; LUAD—S9, S11) were determined using NTA. Bar charts show particles per fraction using percentage (%), particle concentration, and the mode particle size (nanometers, nm) obtained for the pEV samples. Here, adjusted p-values were marked as * < 0.05, **** < 0.0001. (d) The scatter plot shows expression pattern for a panel of 37 EV surface proteins and 2 internal controls (mIgG1, REA), in pEV samples analyzed using a multiplex bead-based EV flow cytometry assay. (e) Representative TEM images show the morphological characteristics of particles present in the pEV samples, enriched from plasma of healthy control and cancer patients using the SEC columns. Scale bar, 500 nm. (f) Western blot was used to investigate the presence of the classical pEV markers, ALIX and FLOT1, and lipoprotein marker, APOA1, in the pEVs. Here, healthy donor pEVs are marked as control (ctrl). Full blots are shown in Figure S2. (g) The stacked bar graph compares the pEV proteome detected in our study within the category indicated by the MISEV 2018 guidelines.

We performed EV isolation using SEC columns starting from only 400 μL of plasma per sample. We visualized the separation and elution of plasma components, i.e., the eluted pEVs and other fractions using Coomassie stained SDS protein gels (Figure S1). To characterize the enriched pEV samples in terms of size, number of particles, and common surface markers, we analyzed pEVs derived from healthy control plasma and cancer patients (N_LUAD_ – 2, N_MM_ – 2) by multiple EV characterization methods including nanoparticle tracking analysis (NTA), multiplex bead-based EV flow cytometry, transmission electron microscopy (TEM), and Western blot. Using NTA, we analyzed the eluted fractions: F1 (pEVs) and the F2 fraction (eluted immediately after the F1; for quality control). NTA analysis showed that the F1 (pEVs) fraction had significantly higher particle percentages (75.0–83.8%) compared to the F2 fraction (16.2–27.3%) in all the samples (Figure 2a). Therefore, only the F1 fraction was selected for further pEV characterization and downstream MS analysis. The concentration of particles was highest in the healthy control pEVs, followed by those in MM and LUAD pEVs (Figure 2b). The particle size distribution ranged from approximately 120 to 175 nm in pEV samples, consistent with the expected recovery range of SEC columns (70 nm to 1000 nm) used for pEV enrichment. The mode size of pEVs was on average 142.8 nm, revealing no significant particle-size difference between the samples (Figure 2c). The particle concentrations of the pEVs varied, yielding an average of 2.02E + 11 particles from 400 μL of plasma input. Particles smaller than 100 nm were detected in low concentrations, indicating a reduced coelution of lipoproteins, which typically measure less than 60 nm^29^ and other non-EV proteins in the EV fractions.

To evaluate the surface protein composition of the particles and confirm the presence of EVs in pEV samples, a multiplex bead-based flow cytometry assay was performed for 37 EV surface proteins simultaneously. In total, 13 different EV surface proteins were present above background levels in all the samples (Figure 2d). Along with the well-established EV marker proteins, CD9, CD63, and CD81, the pEV samples were also positive for platelet associated markers (CD41b, CD42a, and CD62p), suggesting a portion of the pEVs could be of platelet origin.^22^ However, the platelet markers detected by flow cytometry were not detected by SEC–HiRIEF–MS proteomics in pEVs or plasma samples, suggesting they were either not present or in low concentrations below the MS detection limit. The detection of HLA-ABC and HLA-DRDPDQ in all samples indicates the presence of EVs released from the MHC-II class containing cells. The cell-specific proteins such as CD56 - NK cell marker, CD8 - T cell marker, CD44 and CD29 - mesenchymal stromal cell surface marker, were also detected in the pEVs. Transmission electron microscopy (TEM) showed intact vesicles within the expected size range (Figure 2e).

To access contamination from lipoproteins and confirm the presence of EVs at the protein level, we analyzed both pEV and plasma samples for EV and lipoprotein markers using Western blot (Figure 2f). The blot displayed dark protein bands in pEVs for EV markers- ALIX and FLOT1, with very light or no protein bands in plasma. Lipoprotein marker, APOA1 expression was significantly low in pEVs as compared to the plasma samples. Next, we determined the abundance of classical EV markers in pEVs and plasma samples using LC–MS/MS proteomics data. Proteins, including tetraspanins CD151, CD63, CD9, CD81, Flotillin FLOT1, ESCRT-associated PDCD6IP and exosome marker syntenin-1 (SDCBP) were enriched in pEVs as compared to plasma samples. Notably, all above EV marker proteins (excluding CD9, CD81), were detected in pEVs, but not in plasma. This observation suggests that these EV proteins may either be less abundant in the plasma sample types or more difficult to detect by MS. We also assessed the presence of some known unspecific non - pEV proteins that are commonly co-enriched along with EVs, as defined in the MISEV 2018 guidelines.^29^ A comparison with the MISEV protein categories revealed the number and percentage of EV proteins and co-enriched non-EV proteins identified in our pEV proteome (Figure 2g). Here, we found 38 proteins in category 1 (transmembrane or GPI anchored proteins associated with plasma membrane and endosomes), 23 proteins in category 2 (cytosolic proteins recovered in pEVs), 14 proteins in category 3 (major components of non-EV coisolated structures for non-EV proteins), 6 proteins in category 4 (nuclei, mitochondria, ER, Golgi, autophagosomes, and others for enriched specifically in small EVs), and 10 proteins in category 5 (cytokines, adhesion). In the category of Tetraspanins and ESCRT machinery, 25 proteins were identified in the pEVs. In summary, the pEVs isolated with SEC were consistent with size, morphology, and protein composition of EVs.

### HiRIEF MS Method Optimization for pEV Proteome Quantification

**Figure 3:**
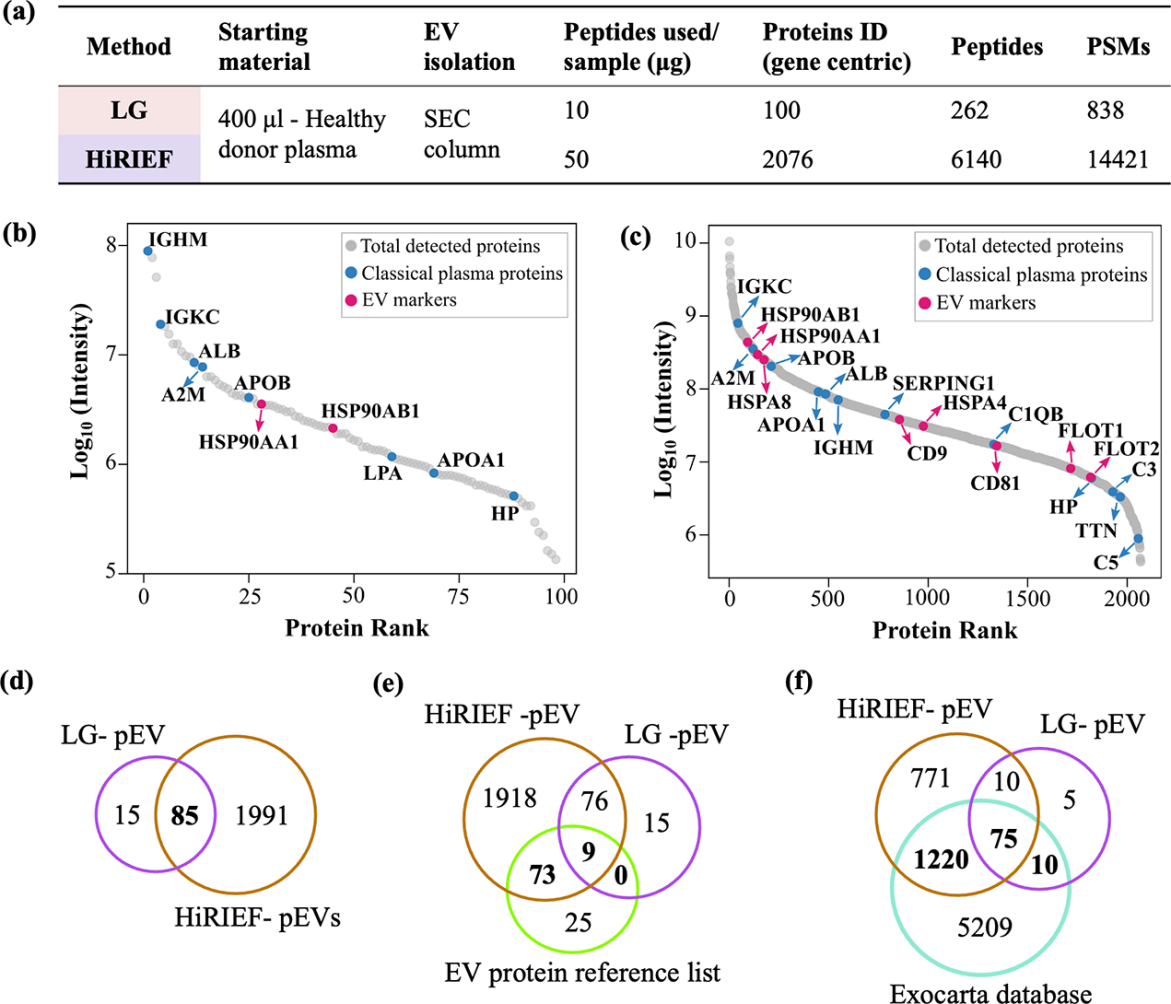
Method assessment for quantitative pEV proteomics using healthy control plasma. (a) Initial MS output summary of the pEV proteomes. The plots show distribution of protein abundance based on MS1 precursor area intensities for the pEV proteome generated by using (b) LG and (c) HiRIEF–MS methods. Here, the classical plasma (blue) and well-established pEV (pink) marker proteins have been specifically indicated by the gene symbol. The Venn diagram shows protein overlap (d) between the LG and HiRIEF–pEV proteomes and (e, f) their overlap with the EV protein reference list and ExoCarta EV database. Here, the ExoCarta database list included only proteins identified in EVs for human species using mass spectrometry.

To evaluate the potential of prefractionation for improved proteome coverage in pEV proteome analysis, we used pEVs isolated from healthy control plasma, as described above. We generated pEV proteomes by applying both conventional LG and HiRIEF–MS methods to the same sample and evaluated their performance based on the proteome coverage and analytical depth. In total, we detected 262 peptides and 100 proteins in the pEVs using LG–MS, and 6140 peptides and 2076 proteins using the HiRIEF–MS method. Though we detected less proteins than expected using the LG approach, the HiRIEF findings clearly reflected the benefit of applying prefractionation prior to MS analysis in pEV proteomics (Figure 3a). To explore proteins detected in each sample, we ranked proteins found in the HiRIEF and LG–pEV proteomes based on the MS precursor area as a measure of the protein abundance, and highlighted the well-established EV markers (pink) and classical plasma proteins (blue)^22,30^ (Figure 3b,c). In total, we found a protein overlap of 85 proteins between the pEV proteomes obtained by using the LG and HiRIEF–MS methods (Figure 3d).

In-depth proteome profiling of human pEVs using SEC–HiRIEF–MS allowed us to detect several well-established EV markers and EV-specific proteins, including tetraspanins, chaperones, annexins, flotillins, and several most frequently observed proteins associated with the extracellular exosome, extracellular region, and endosomal trafficking^2,5^ (Figure S3a). In a Gene Ontology (GO) analysis, both LG and HiRIEF–pEV proteomes showed enrichment for *extracellular exosome* in the GO-cellular component (CC) category (Figure S3b,c). In addition, a comparison with the “EV protein reference list” of 107 most observed EV proteins including “Top-100 proteins” of the ExoCarta database^7^ and 13 novel pan-EV marker proteins in humans, as reported by Hoshino et al.,^5^ showed a 77% overlap for the HiRIEF–pEV proteome and 8% overlap with LG pEV proteome (Figure 3e, Datasheet S1). The LG and HiRIEF–pEV proteomes also shared 85 and 1295 proteins, respectively, with the ExoCarta EV database (Figure 3f). In summary, the above comparisons indicate that the prefractionation method facilitates comprehensive proteomics exploration by increased detection of EV-specific proteins in pEVs.

### Parallel Quantitative Proteomic Analysis of Plasma and pEVs Derived from Metastatic Cancer Patients

One important aspect in plasma biomarker discovery is the co-occurrence of both soluble proteins and pEV proteins, both of which could harbor information about the disease state. When enriching for EVs from human plasma, soluble plasma proteins are commonly co-isolated during the experimental procedure. The pEV proteome will therefore contain both soluble plasma proteins and true EV proteins. Similarly, the plasma proteome will inevitably include pEV proteins (unless specifically removed), albeit at a low concentration. In such a scenario, identifying the true EV protein composition or confirming which of the proteins detected in the pEV samples are actually present in EVs and not carried over from the plasma, as well as specific biological contributions made by the pEVs, is difficult. The lack of total plasma reference sample in previously conducted global MS-based EV studies limits the potential of showing the benefit of the plasma EV enrichment as well as the contribution of the soluble proportion of the plasma proteome.

To fill this knowledge gap, we conducted a small proof-of-concept study by performing the parallel comprehensive proteome quantification of both plasma and pEV samples from the same patients. This approach aimed to better understand the contribution of pEVs to the global plasma proteome and explore unique proteins present in each compartment to evaluate the potential of pEV proteomics in detecting disease-specific EV proteins and identifying biomarkers using samples from metastatic LUAD and MM patients.

### Comparative Proteomic Analysis of Cancer Plasma and pEV Proteomes

**Figure 4:**
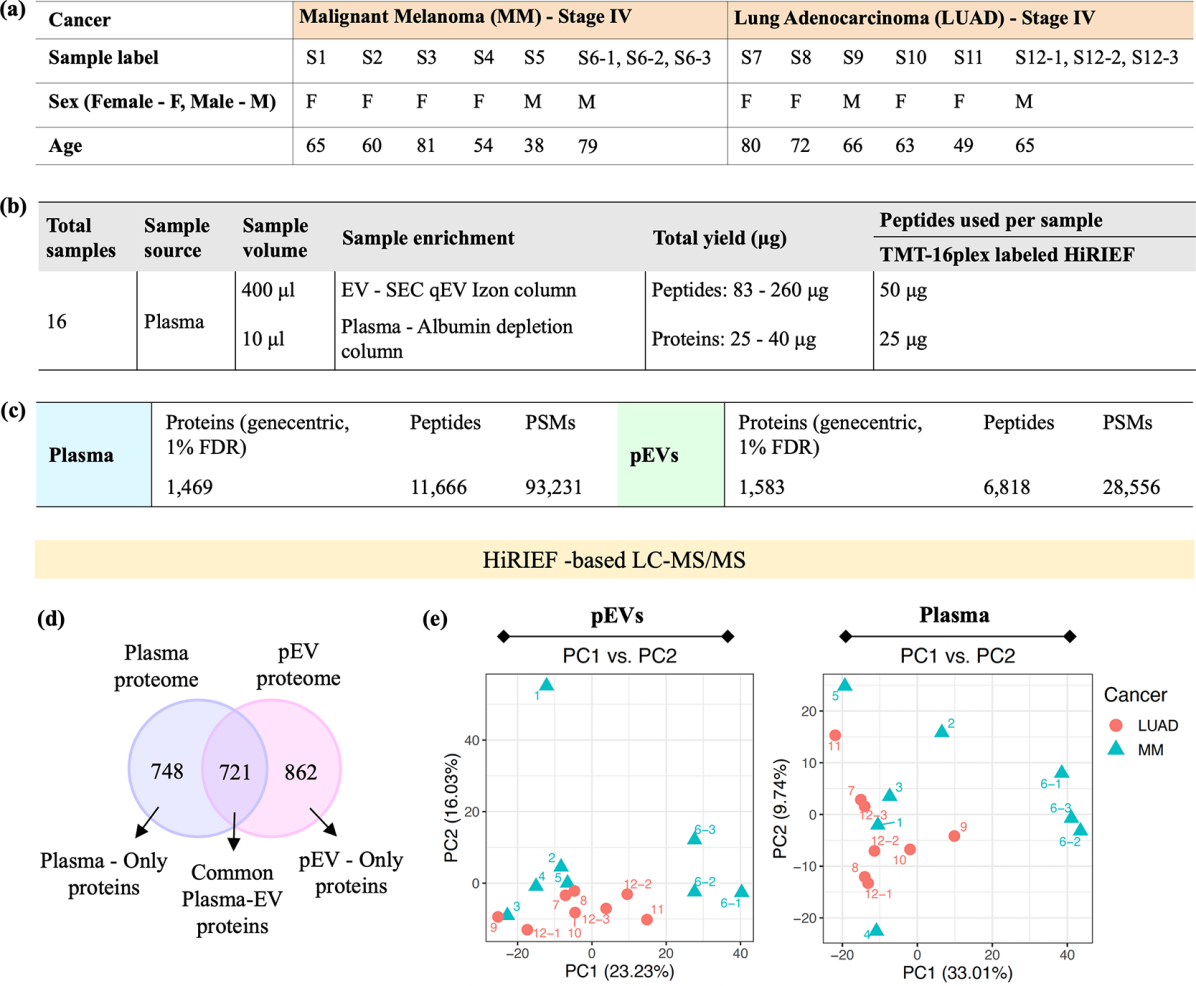
Quantitative proteomic profiling of pEVs and plasma taken from MM and LUAD cancer patients. (a) Sample layout for cancer patients. Here, we have taken experimental triplicates for S6 and S12 samples. (b) Experimental details for the study. (c) MS output summary of the cancer pEV and plasma proteomes generated using the SEC–HiRIEF–MS analysis. (d) Shows protein overlap for total proteins identified and (e) PCA analysis showing variance distribution (%) for PC1 versus PC2, in the HiRIEF–pEV and plasma proteomes, respectively.

To evaluate the performance of the SEC–HiRIEF–MS workflow setup for pEV proteomics, six LUAD samples, six MM samples, and experimental triplicates for the S6 and S12 patients, i.e., a total of 16 pEV samples, were analyzed in parallel with 16 plasma samples from the same patients (Figure 4a,b). We used two TMT16-plex sets to analyze all pEV and plasma samples separately within each TMT set, which improved the overlap in proteins identified between samples. With SEC–HiRIEF–MS, we could identify and quantify a total of 6818 unique peptides, 28,556 PSMs and 1583 proteins in the cancer pEVs, and 11,666 unique peptides, 93,231 PSMs and 1469 proteins in the cancer plasma, across all the samples (Figure 4c). Heatmap depicting the expression of total proteins identified in both plasma and pEVs using SEC–HiRIEF–MS is shown in Figure S4. The protein overlap of plasma and pEV proteome shows that 862 proteins were detected only in the pEVs, 748 proteins were detected only in the plasma proteome, and 721 common proteins were present in the plasma and pEV proteomes (Figure 4d). The variance in protein quantities across all samples was evaluated with principal component analysis (PCA). The PC1 and PC2 components effectively separated the LUAD and MM samples for both sample types: pEV and plasma (Figure 4e). The S1-MM sample appeared as an outlier, accounting for a large part of the variance in PC2. The S6-MM and S12-LUAD sample triplicates clustered closely together for both pEV and plasma proteome analyses. However, we observed intraspecimen variability among the triplicates, indicating a need for improvements in sample preparation and MS method optimization to achieve higher reproducibility. In future validation studies, increasing the sample size could provide tight clusters, further improving the distinction between the two groups and validating the observed separation.

### Enrichment of EV Specific Proteins in Cancer Plasma and pEVs

**Figure 5:**
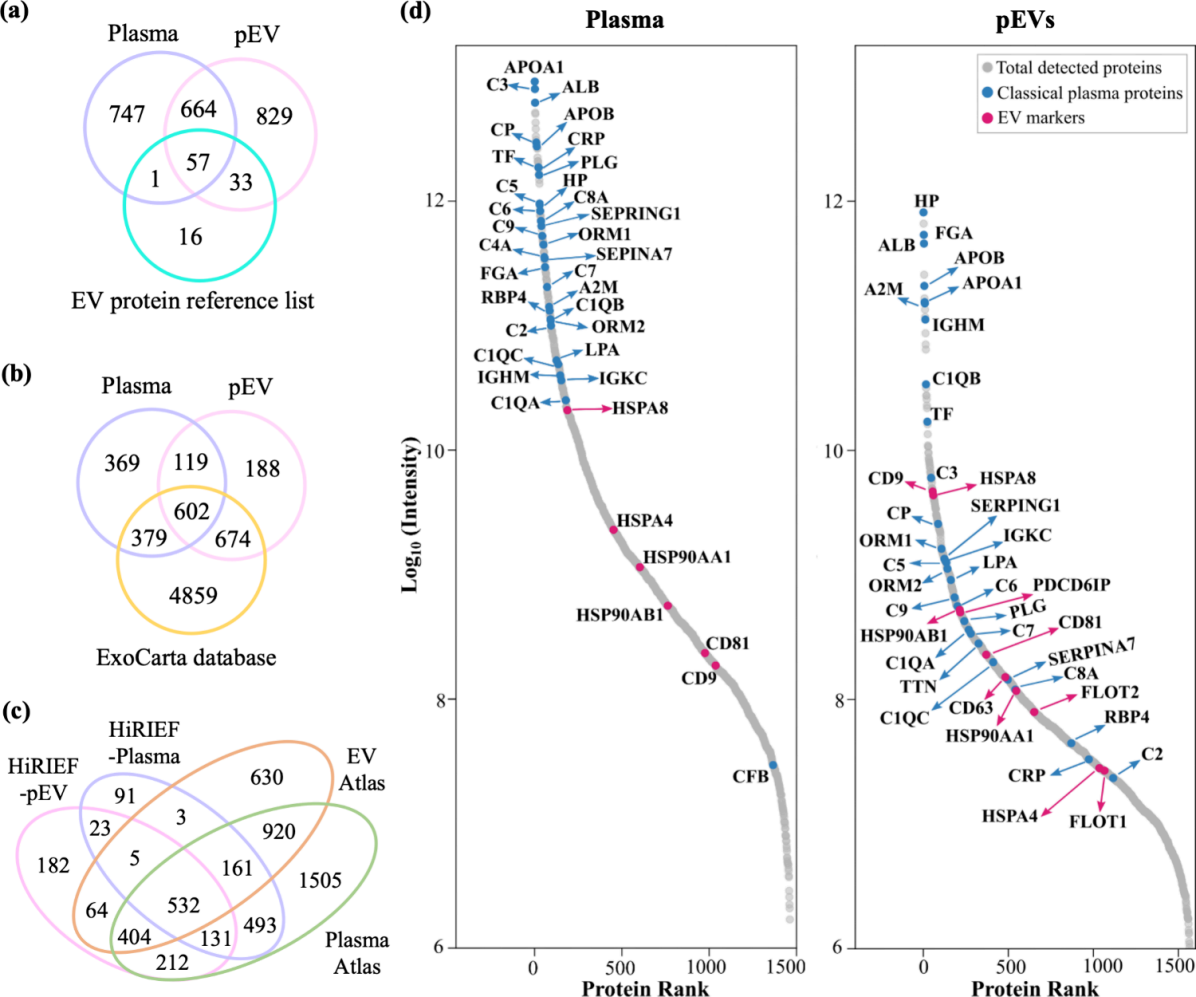
Distribution of protein abundances and overlap of cancer patient-derived plasma and pEVs with the publicly available plasma–EV databases. (a–c) The Venn diagrams show protein overlap between the plasma and pEV proteins detected using the HiRIEF with the EV protein reference list, ExoCarta EV database, and Plasma–EV proteome from the PeptideAtlas. (d) Distribution of protein abundances based on MS1 precursor areas in the HiRIEF–plasma and −pEV proteomes from metastatic LUAD and MM patients. Here, classical plasma proteins and well-established EV marker proteins are highlighted in blue and pink, respectively.

To evaluate the presence of EV specific proteins, the plasma and pEV proteomes were first compared with the EV protein reference list (Datasheet S1). In the pEVs, 90 proteins from the protein reference list were detected (84% of the EV reference list), and in the plasma proteome, 58 proteins were identified (54% of the EV reference list) (Figure 5a). Comparison to the ExoCarta database revealed an overlap of 1276 proteins with the pEV proteome and 1469 proteins with the plasma proteome, respectively (Figure 5b). In addition, plasma and pEV proteomes were compared with the “Human plasma and EV proteome” databases provided by the PeptideAtlas MS data repository.^27^ In the PeptideAtlas data sets, 74% of the proteins present in their EV proteome database (n = 2750 proteins) were also quantified in their plasma proteome database (n = 4389 proteins), while in our HiRIEF data sets, only 46% of the pEV proteome was shared with the plasma proteome, showing a more distinct separation between the plasma and pEV proteome data set generated in our study (Figure 5c). The presence of these most frequently observed EV proteins in both plasma and pEVs, brings attention to several aspects. First, global in-depth plasma proteome analysis would also detect some EV-specific proteins, however, separate pEV proteomic analysis is required to obtain a comprehensive pEV proteome. Second, the detection of several EV specific proteins in the plasma proteome suggests the need for more reliable and stringent definitions of EV marker proteins than the “most frequently observed proteins.”

In the pEV proteome, the distribution of protein abundances based on MS1 precursor area shows few classical plasma proteins (highlighted in blue)^30^ and several conventional pEV markers (highlighted in pink)—CD9, HSPA8, HSP90AB1, ALIX/PDCD6IP, CD81 and CD63—detected among the highly abundant proteins, confirming the findings from the initial EV enrichment analysis above (Figure 5d, right). CD9, CD63, and CD81 detected in pEVs using the SEC–HiRIEF–MS approach were also found in the flow cytometry data analysis (Figure 1). In addition, many other established EV markers—HSP90AA1, FLOT2, HSPA4, and FLOT1—were detected in the medium abundance range. A similar protein distribution plot of the plasma proteome shows several classical plasma proteins present among the highly abundant proteins, with only a few EV marker proteins detected in the medium abundance range (Figure 5d, left). As evident from the distribution plot, many of the classical plasma proteins were detected in both plasma and pEV proteomes; however, the protein abundance of these classical plasma proteins was greatly reduced in the pEV proteome.

In the current study, we applied SEC for pEV enrichment with the goal of covering the entire circulating EV population in plasma, rather than focusing on enrichment of specific EV subtypes. So, we further mined the cancer plasma and pEV proteomes to extract proteins detected using this strategy that are primarily associated with EV cargo selection, ESCRT complexes, EV trafficking/sorting, tetraspanins, chaperones, EV biogenesis, nuclear-cytoplasmic, RNA/DNA binding proteins, enzymes, integral plasma membrane, and extracellular space^1^ (Table 1). Due to their unique distribution across EVs, many of these proteins are also used to define EV subpopulations such as exosomes, small-sized EVs (sEVs) and large-sized vesicles.^22,29,31^ The majority of these proteins were detected exclusively in the pEV proteome (Table 1), suggesting that they were either absent or present in low abundance in plasma samples, making them insufficient for MS detection. The SEC–HiRIEF–MS workflow allowed us to detect and quantify more unique proteins in pEVs, which are often missed in the plasma proteome due to their low relative abundance.

**Table 1:** List of EV-Specific Proteins Identified in EVs Enriched from Cancer Patient Plasma, Using the SEC–HiRIEF–MS Workflow^a^

Enriched proteins in EVs	Protein name
ESCRTs	VPS37B, VPS37C, CHMP4B, MVB12B, CHMP2A, PLSCR1, *VPS29*
ESCRT accessory	VTA1, PDCD6IP (ALIX)
Tetraspanins	CD63, CD82, CD151, TSPAN4, TSPAN8, TSPAN9, TSPAN14, **CD9, CD81**
SNARE’s	STX2, **STX3**, STX4, STX7, STX11, SNAP23, VTI1B, VAMP8
RNA binding proteins	RPL13, RPL13A, RPS25, CPNE1, CPNE3, HNRNPA2B1, **ANXA2**, ANXA3, ANXA4, ANXA5, ANXA6, ANXA7, ANXA11, **HNRNPC**, **HNRNPK**, HNRNPM, HNRNPR, **EEF2**, **HMGB2**
Cargo selection	VPS26A
Trafficking/sorting	SDCBP, ARRDC1, RAB1A, RAB1B, RAB5B, RAB8A, RAB8B, RAB11B, RAB2A, RAB7A, RAB10, RAB14, RAB18, RAB35, FLOT1, FLOT2, **CD44**
EV biogenesis	ARF6, PLSCR1, PLSCR3, **CAPN1**, **CAPN2**, CAPN5
Integral membrane proteins	CKAP4, **ITGAV**, **ADAM10**, **ADAM17**, **ADAMTS13**, *ADAM9*, *ADAMTS1*, *ADAMTS2*, *ADAMTS8*, FASN, ITGA2B, ITGA4, ITGA6
Mitochondrial Proteins	VDAC1, VDAC2, PHB2, **SLC25A5**, **SLC25A24**, **SLC25A11**
Cytoskeleton/microtubule	MYO1C, MYO1G, MYO6, **TPM1**, FLNB, KIF2A, KIF5A, MYH10, MYH14, TUBA4A, IQGAP1, IQGAP2, **EZR**
Enzymes	ATP5F1C, ATP5IF1, ATP5PD, **GAPDH**, **ACLY**, **ENO1**, **CASP14**
Chaperones	CSE1L, **YWHAB**, **YWHAE**, **YWHAG**, **YWHAH**, **YWHAQ**, **YWHAZ**
ABC transporters	ABCB4, ABCB11
Heat shock proteins	**HSP90AA1**, **HSP90AB1**, **HSPA4**, **HSPA8**

a Proteins detected in both plasma and pEVs (text in bold) and proteins detected only in plasma proteome (text in italics).

### Differentially Expressed Proteins Identified in Cancer Plasma and pEVs

**Figure 6:**
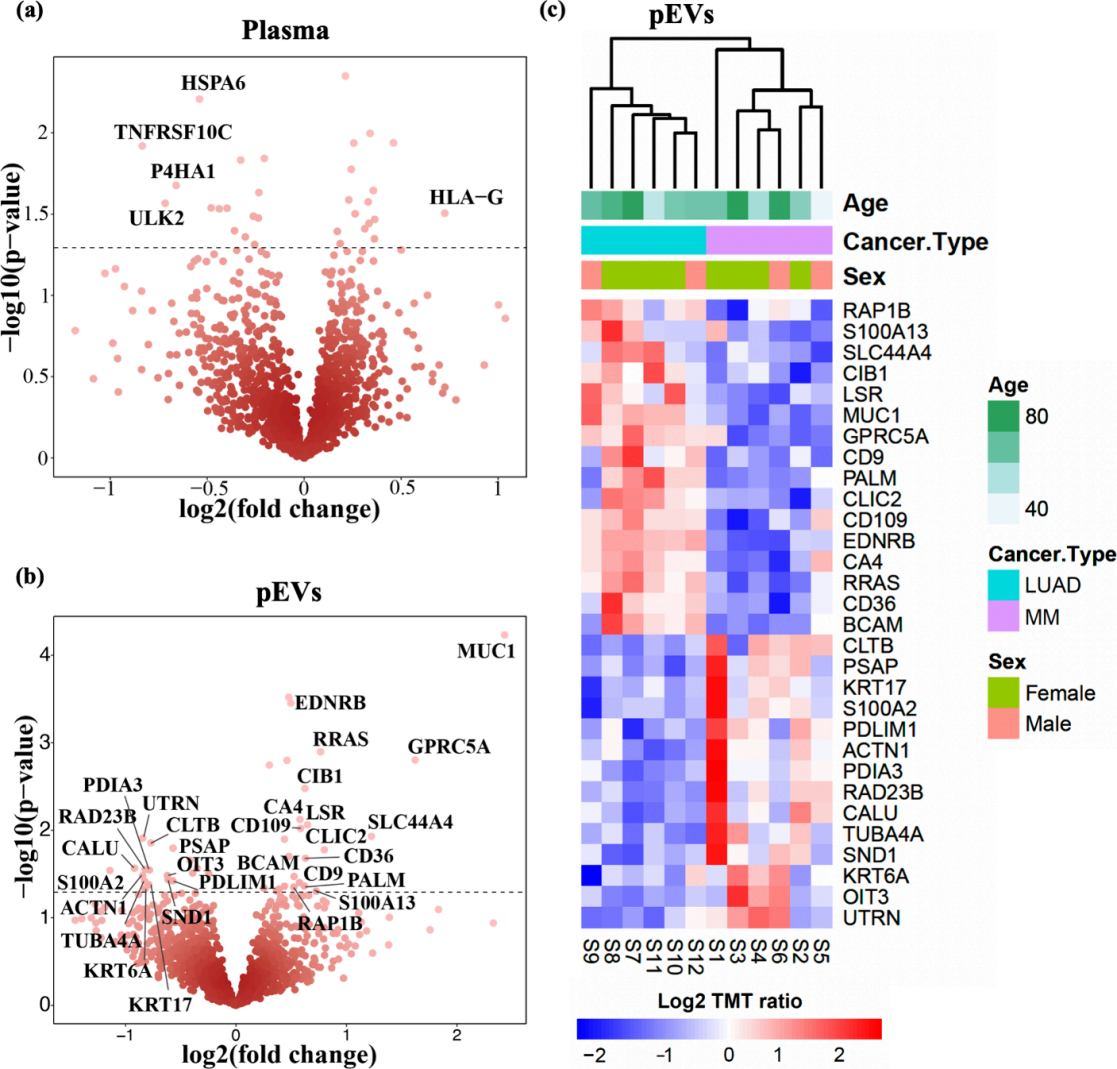
Differentially expressed proteins identified in the cancer patient plasma and pEVs with respect to cancer type. (a, b) The volcano plots show DEPs (left, down-regulated; right, up-regulated) for metastatic LUAD vs MM, identified in the plasma and pEV proteome generated using SEC–HiRIEF–MS. Statistical significance with a p-value cutoff <0.05, ranging between 0.05 and 0.00006 (Table S1). (c) Heatmap representation and hierarchical clustering of the DEPs detected in the HiRIEF–pEV proteome. The data are centered, and scaling is applied to proteins. The samples were clustered using the Euclidean distance and average linkage clustering.

To test if the EVs enriched from plasma of LUAD and MM patients contain proteins derived from the disease, we performed differential analysis in plasma and pEVs for LUAD and MM cancer group comparisons using the *t* test and selecting proteins with a 0.5-fold or larger increase and a p-value of <0.05 (Figure 6a,b). Applying these criteria, we have identified a total of 30 differentially expressed proteins (DEPs) in the cancer pEV proteome and only 5 DEPs in the plasma proteome (Table S1). Hierarchical clustering of protein abundance for DEPs identified in pEVs shows a clear stratification of samples based on the cancer type (Figure 6c). Many of these DEPs have been suggested as potential clinical prognostic markers, due to their functional association with tumor growth and progression.^32−37^ We found upregulation of MUC1 and RAP1B in LUAD pEV samples compared with the MM pEV samples. MUC1 is known to be highly expressed in epithelial cancers^38^ and increased RAP1B expression has been associated with poor prognosis in late stage LUAD patients.^39^ In contrast, the protein disulfide isomerase A3 (PDIA3) and keratins KRT6A and KRT17 were upregulated in the MM pEV samples compared to the LUAD pEV samples. PDIA3 has been recently suggested as a robust prognostic biomarker for pan-cancers and could significantly predict anti-PDL1 therapy response.^40^ Keratins KRT6A and KRT17 have also been suggested as potential prognostic biomarkers for melanoma due to their role in cancer cell proliferation, immune cell infiltration and metastasis.^41^ In conclusion, the findings suggest that EVs provide unique additional proteomics information that cannot be obtained from plasma alone and that proteins of EV origin form an integral part of the plasma proteome. The findings support the applicability of prefractionation-based MS analysis for comprehensive pEV proteomics and the potential in biomarker discovery to detect disease-derived pEV proteins.

## Discussion

Plasma-based liquid biopsies are highly desirable for disease diagnosis, prognosis, and treatment outcomes, as they can potentially monitor molecular events taking place in any tissue in the entire body. However, the human plasma proteome exhibits high dynamic range and variability in terms of proteins concentration. The top abundant plasma proteins hamper the measurement of the hundreds of low abundant proteins, which primarily includes tissue-leakage proteins and signaling molecules that are potential disease markers critical to the clinical biomarker discovery applications^30^ and thereby limiting the analytical depth. As a result, there has been an increased interest in exploring circulating EVs enriched from plasma of patients with pathological conditions in biomarker discovery studies, where the pEVs may contain relevant protein markers for disease progression and treatment response.^2^ Here, we present the SEC–HiRIEF–MS workflow for deep and quantitative proteomic profiling of pEVs, highlighting their potential in clinical biomarker discovery and the detection of disease-specific proteins. Advanced prefractionation-based MS methods developed to increase the proteome coverage in complex samples, such as cells or tissues, have not yet been applied to pEV proteomics. We suggest that the SEC–HiRIEF–MS approach could significantly improve pEV proteome coverage. Recent advances in DIA–MS methods display improved protein detection rate and applicability in clinical proteomics but face challenges with data incompleteness and high missing values;^42,43^ this limitation hinders the quantitative analaysis of significant protein alterations across entire cohorts. In contrast, the HiRIEF prefractionation approach overcomes these limitations, providing comprehensive protein identification and quantification across all samples with better accuracy and sensitivity. This makes HiRIEF advantageous for detailed proteome quantification and individual patient- and group-level comparisons in clinical cohorts. The advanced HiRIEF workflow has trade-offs in terms of reproducibility due to its complex workflow and need for method optimization. However, by leveraging the strengths of HiRIEF, we can achieve more detailed and accurate proteome identification and quantification in pEVs, enabling better sample comparisons, detection of disease-specific proteins, and advancing pEV proteomics in clinical research.

In our study, the SEC–HiRIEF–MS workflow allowed us to quantify the low-abundance proteins and noncanonical proteins in the pEV proteome by increasing the analytical depth. Starting with just 400 μL of plasma per sample and without any extensive EV enrichment strategies, we have identified and quantified more than 2000 proteins in pEVs for all of the samples. Comparative analysis of control pEV proteomes generated using conventional LG– and HiRIEF–MS, showed significantly enhanced protein identification and EV protein marker abundance in the pEV proteome detected using the SEC–HiRIEF–MS approach. In addition, we have shown that by employing prefractionation we have improved the detection of EV markers, such as CD9, CD63, CD81, FLOT1/2, and ALIX/PDCD6IP, and identified more EV-specific proteins in the pEV proteome than previously published EV studies.^44,45^

In addition to significant improvement in EV protein quantification, our study provides a comprehensive analysis of plasma and pEV proteomes in parallel, yielding novel insights into the soluble and EV protein compartments of plasma from cancer patients. Recently, Lattman et al. (2024)^46^ also made efforts to address this gap by comparing the proteome of plasma-derived EVs and total plasma from the same samples. Remarkably, previous pEV studies have largely overlooked the dynamics of interaction between pEVs and the soluble plasma protein compartment. Typically, these studies isolate pEVs from plasma without simultaneously assessing the soluble plasma component, thereby limiting the clinical insights that parallel plasma and pEV analyses could provide. Analyzing only pEVs makes it challenging to identify proteins specific to the pEV proteome, emphasizing the necessity of analyzing the whole plasma proteome in parallel for accurate comparisons. In our study, SEC–HiRIEF–MS approach allowed us to improve both protein identification and quantification in pEVs from plasma of LUAD and MM patients, as compared to previous melanoma and other cancer proteomic studies of pEVs.^22,47^ It is important to note that the experimental and technical differences limit direct comparability between our study and the previous investigations. Studies by Kalra et al.,^48^ de Menezes-Neto et al.,^49^ and Hoshino et al.^5^ identified EV proteins in human pEVs, using large plasma sample volumes, sequential ultracentrifugation-based EV isolation, and the conventional LG–MS method. Others used immuno- or affinity-enrichment-based methods for enrichment of specific EV subpopulations and not total EV isolation. For instance, the studies by Karimi et al. and Muraoka et al. performed pEV proteomics for selectively enriched EV subpopulations using tetraspanin- and phosphatidylserine-targeted assays, from plasma/serum within a small cohort of six healthy individuals. Additionally, although these studies provide good qualitative data, they lack further quantitative or comprehensive EV proteome analysis.

Comparisons between the plasma and pEV proteome showed a high degree of overlap that is due to the co-enrichment of plasma proteins during EV isolation, and it is clear that not all proteins detected in the pEVs are EV proteins. Similarly, we also detected several classical pEV proteins in the plasma proteome; however, their quantification is hindered due to the presence of highly abundant plasma proteins. Hence, the detected EV protein cargo is represented by the unique EV-associated proteins and the common protein cores found in plasma and pEVs. The unique EV-associated proteins detected in this study include several conventional EV markers, proteins associated with EV biogenesis, EV surface, EV sorting/transport, and many cancer-related proteins (such as RAP1B, FLOT1/2, Annexins, RAB proteins, SLC2A1, SLC7A5, PSMD1, CKAP4, LEF1, PAX5, and TCL1A), which were not captured in the plasma proteome or when performing conventional LG LC–MS/MS analysis. Comparison of pEVs from LUAD and MM patients revealed differential expression of several proteins previously reported to be associated with metastatic LUAD and MM, such as MUC1, SLC44A4, OIT3, PDIA3, KRT17, and KRT6A. EV proteins have previously been shown to strongly influence nonsmall cell lung cancer (NSCLC) metastasis by regulating tumor cell invasion, proliferation, angiogenesis and immune suppression.^50,51^ Similarly, MM-derived exosomes contain specific protein cargo that seems to promote tumor growth and immune escape mechanisms.^52,53^ Here, we also found more significantly differentially expressed proteins detected in pEVs for LUAD versus MM compared with the total plasma analysis, reaffirming the complementarity of both plasma and pEV profiling in biomarker discovery. In conclusion, our study underscores the potential of identifying disease-specific proteins within pEVs and conducting comprehensive pEV proteomic analyses using a prefractionation-based LC–MS methodology. Although our investigation was conducted within a small cohort comprising LUAD, and MM patients, it sheds light on the feasibility of this approach for broader clinical applications and how prefractionation-based LC–MS approach could further benefit comprehensive pEV proteomics studies.

Based on our study findings, several potential future research directions emerge to further advance pEV proteomics. Our study uses HiRIEF for its advantages over other methods and its proven potential in clinical proteomics. However, it would be valuable to evalute other prefractionation-based LC–MS approaches for pEV proteomics to determine their effectiveness in achieving high proteome coverage. Future studies could optimize and compare various prefractionation methods for better protein identification and quantification, potentially leading to more accurate pEV proteomics and biomarker discovery. Given the crucial role of pEVs in exploring the therapeutic potential across various pathological conditions, prefractionation approaches can also be applied to other biomarker studies. Additionally, further exploration of the complementarity between plasma and pEV proteomes is essential. Our study demonstrates the importance of parallel proteome profiling of pEVs and plasma for biomarker discovery and shows the value of deep pEV proteome detection and quantification. Investigating the unique and overlapping protein profiles of plasma and pEVs in patient cohorts across diverse conditions could provide deeper insights into disease mechanisms and progression. Comprehensive pEV proteome analysis to delineate the EV protein cargo and associated molecular pathways has a strong potential for identifying circulating biomarker signatures for diagnosing and managing diseases such as cancer.

## Data Availability

All data generated or analyzed during the study, are either included in the article or uploaded as supplementary data. MS raw data have been uploaded to the PRIDE repository via ProteomeXchange with accession number PXD039338 and PXD038528.

## Supporting Information Available

Figure S1. Characterization of depleted plasma and plasma-derived EVs (pEVs) using Coomassie-stained SDS-PAGE analysis of pEVs and depleted plasma protein lysates. Figure S2. Full blots of Western analyses for pEVs and plasma. Figure S3. Enrichment of EV proteins in the pEV proteome of the healthy control plasma. Figure S4. Heatmap representation of the cancer plasma and pEV proteomes. Table S1. List of differentially expressed proteins (DEPs) identified in cancer pEVs for LUAD vs MM cancer type. (PDF)
Datasheet S1. Separate data sheets for EV protein reference list, and its overlap with control pEV–LG and HiRIEF proteome, and cancer pEV and plasma proteomes. (XLSX)
Datasheet S2. Description of HiRIEF gradient scheme. (XLSX)
