# Supplementary material for: Defining the Soluble and Extracellular Vesicle Protein Compartments of Plasma Using In-Depth Mass Spectrometry-Based Proteomics
Source: J Proteome Res. 2024 Aug 14;23(9):4114–27. doi: 10.1021/acs.jproteome.4c00490 (PMC11385381; doi:10.1021/acs.jproteome.4c00490)
Supplement: Supplementary file 1 — pr4c00490_si_001.pdf [file pr4c00490_si_001.pdf]

Defining the Soluble and Extracellular Vesicle Protein Compartments of Plasma Using In-depth Mass Spectrometry-based Proteomics

Nidhi Sharma[1,*], Silvia Angori[1], AnnSofi Sandberg[1], Georgios Mermelekas[1], Janne Lehtiö[1], Oscar P.B. Wiklander[2,3], André Görgens[3,4], Samir El Andaloussi[3], Hanna Eriksson[1,2*,#], Maria Pernemalm[1*,#]

[1]Department of Oncology-Pathology, Science for Life Laboratory, Karolinska Institute, Tomtebodavägen 23, 171 65 Solna, Sweden.

[2]Theme Cancer, Skin Cancer Center, Karolinska University Hospital, Solnavägen 1, 171 77 Solna, Sweden.

[3]Biomolecular Medicine, Clinical Research Center, Department of Laboratory Medicine, Karolinska Institute, Visionsgatan 18, 171 76 Solna, Sweden.

[4]Institute for Transfusion Medicine, University Hospital Essen, University of Duisburg-Essen, 45141 Essen, Germany

[#]shared last authors

*Corresponding authors

Nidhi Sharma, Department of Oncology-Pathology, Science for Life Laboratory, Karolinska Institute, Tomtebodavägen 23, 171 65 Solna, Sweden. Email: nidhi.cct@gmail.com

Dr. Hanna Eriksson, Department of Oncology-Pathology, Karolinska University Hospital, Solnavägen 1, 171 77 Solna, Sweden. Email: hanna.eriksson.4@ki.se

Maria Pernemalm, Department of Oncology-Pathology, Science for Life Laboratory, Karolinska Institute, Tomtebodavägen 23, 171 65 Solna, Sweden. Email: maria.pernemalm@ki.se

Table of Contents

Figure S1. Verification of depleted plasma and plasma derived EVs (pEVs).

Figure S2. Full blots of western analyses for pEVs and plasma.

Figure S3. Enrichment of EV proteins in the pEV proteome of the healthy control plasma.

Figure S4. Heatmap representation of the cancer plasma and pEV proteomes.

Table S1. List of differentially expressed proteins (DEPs) identified in cancer pEVs for LUAD vs. MM cancer type.

Following supporting information files (XLSX) are provided as separate files.

Datasheet S1. Separate data sheets for EV protein reference list, and its overlap with control pEV– LG and HiRIEF proteome, and cancer pEV and plasma proteomes (XLSX).

Datasheet S2. Description of HiRIEF gradient scheme (XLSX).

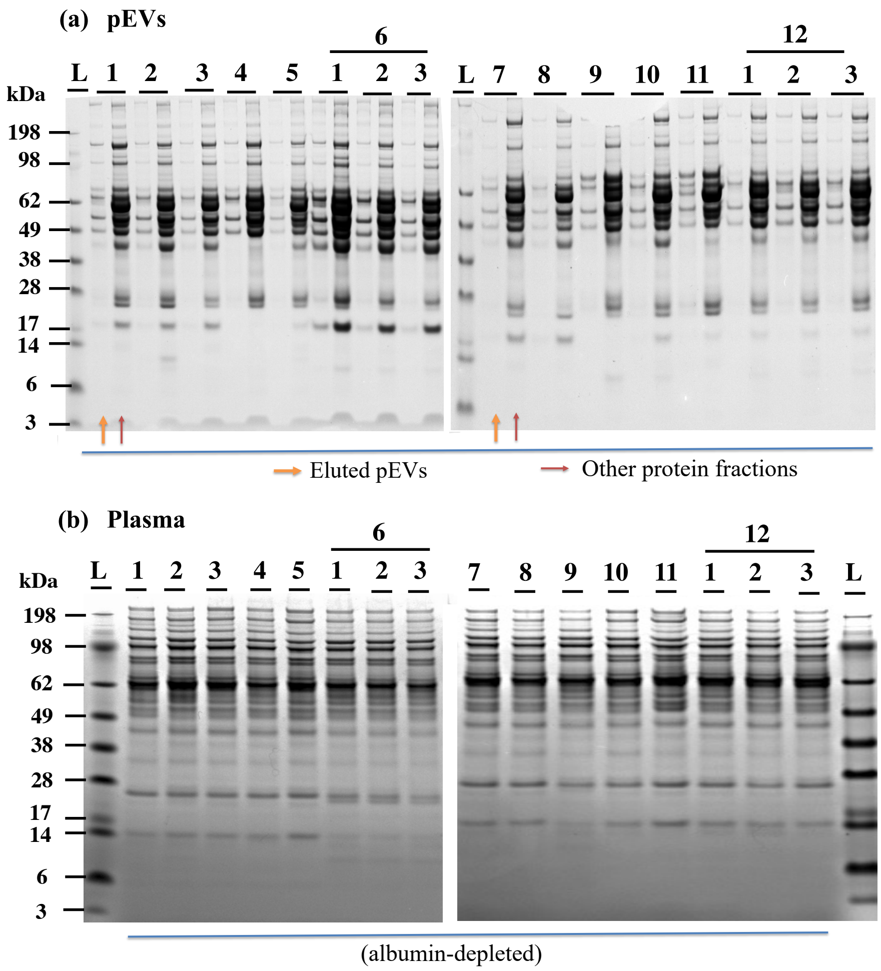

Figure S1. Verification of depleted plasma and plasma derived EVs (pEVs). The figure shows Coomassie-stained SDS–PAGE of pEVs and depleted plasma protein lysates. (a) The gel for pEVs has two wells/lanes for each sample, first lane – pEVs and second lane – pooled other plasma protein fractions eluted from SEC column. (b) The depleted plasma samples show very thin albumin bands. Here, standard protein ladder (L) was used as control. The gel column corresponds to all samples labeled from 1-12 (S1 - S12), including sample triplicates of S6 - 1,2,3 and S12 -1,2,3.

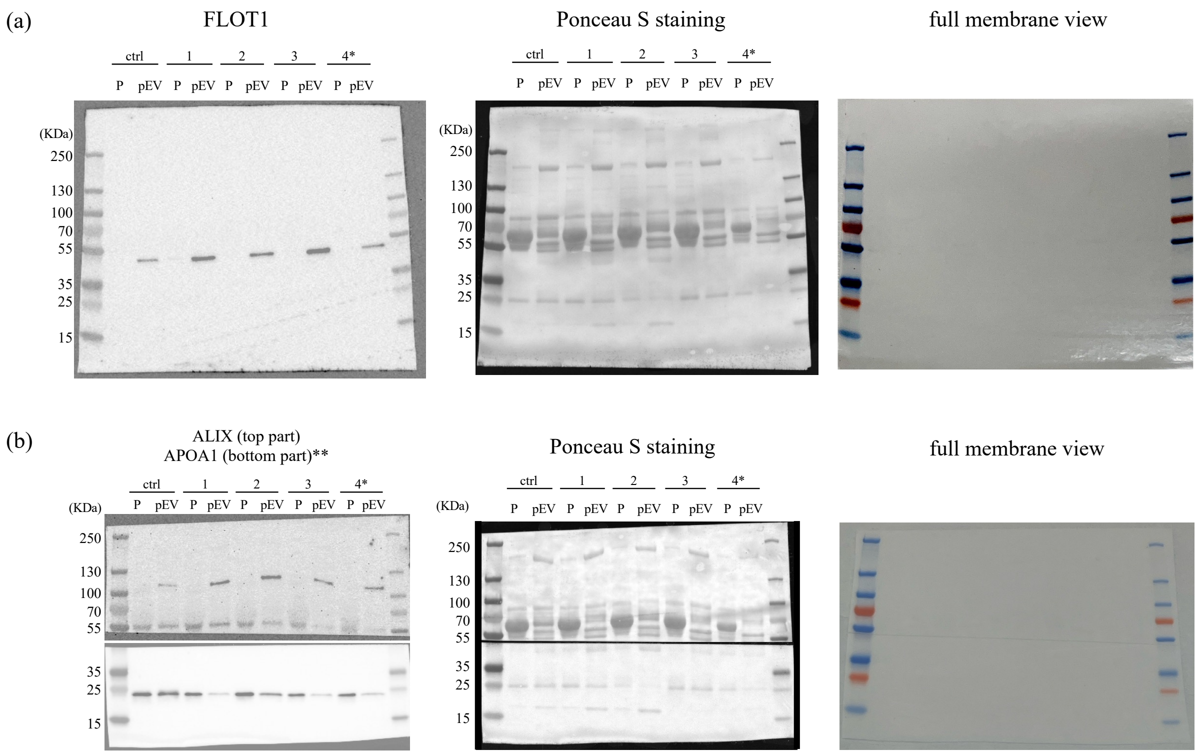

Figure S2. Full blots of western analyses for pEVs and plasma. (a) shows FLOT1 and (b) shows blots for ALIX1 and APOA1. We cropped the membrane to detect and stain for ALIX (top part) and APOA1 (bottom part) proteins, due to their distinctively large size difference. Here, we are showing the two full stained pieces next to each other. The Ponceau S staining was performed after the ALIX/APOA1 staining to avoid interferences with the binding of the primary antibodies.

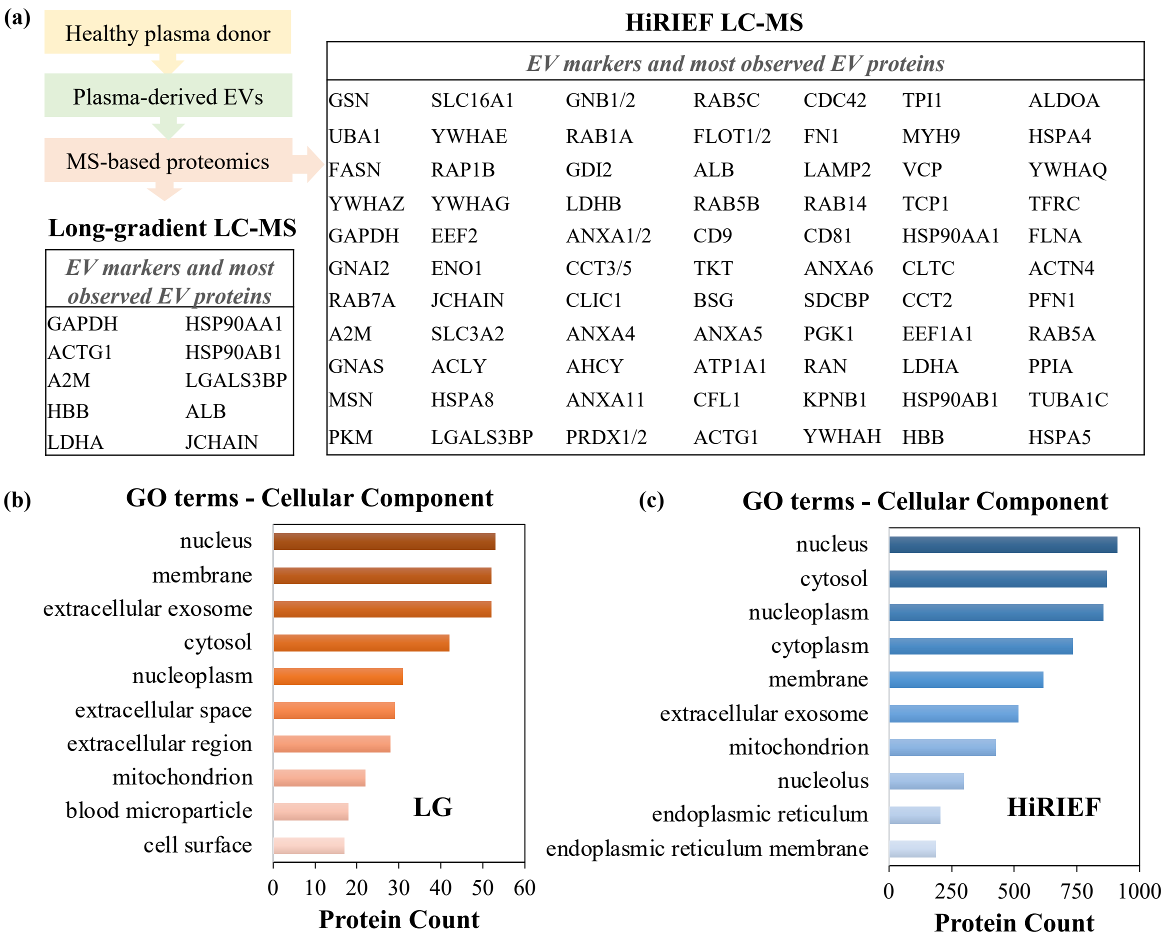

Figure S3. Enrichment of EV proteins in the pEV proteome of the healthy control plasma. (a) The flowchart shows list of well-established EV markers and most frequently observed EV proteins (based on EV protein reference list) detected in the normal pEVs, by applying LG– and HiRIEF– MS methods. Bar charts show gene Ontology (GO) – cellular component (CC) terms distribution for proteins detected in normal pEVs using (b) LG– and (c) HiRIEF– MS methods. We have used DAVID tool to determine the most enriched GO terms. Here, the 10 most significantly enriched terms based on high protein count are displayed.

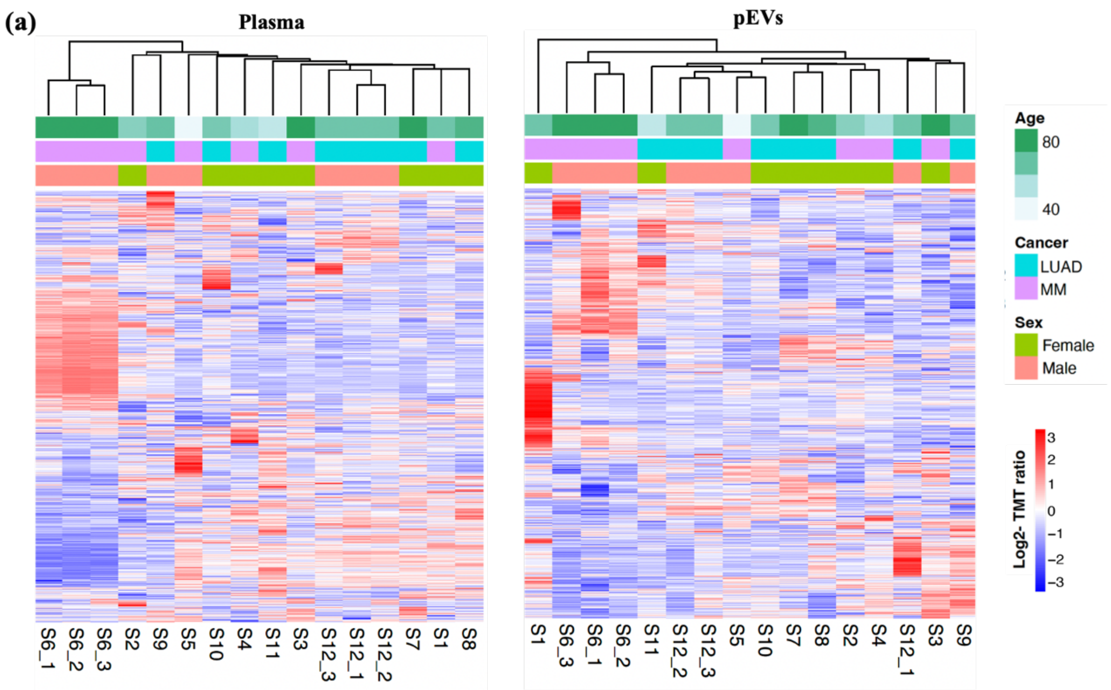

Figure S4. Heatmap representation of the cancer plasma and pEV proteomes. The heatmaps show protein abundance in metastatic MM and LUAD patient (a) plasma and pEVs achieved using the TMT-labeled SEC−HiRIEF−MS workflow. The data was centered, and scaling was applied to proteins. The samples were clustered using Euclidean distance and average linkage clustering methods.

Table S1. List of differentially expressed proteins (DEPs) identified in cancer pEVs for LUAD vs. MM cancer type. Significance cut-off p-value < 0.05.

Up - Regulated Proteins				
**Gene symbol**	**Description**	**Cellular Localization**	**Fold change**	**P-value**
MUC1	Mucin 1	Transmembrane	2.43	0.00006
GPRC5A	G Protein-Coupled Receptor Class C Group 5 Member A	Transmembrane	1.62	0.0016
SLC44A4	Solute Carrier Family 44 Member 4	Transmembrane	1.23	0.012
CLIC2	Chloride Intracellular Channel Protein 2	Transmembrane	0.80	0.0167
RRAS	Ras-related protein R-Ras	Cell Membrane	0.76	0.0012
S100A13	S100 Calcium-Binding Protein A13	Nucleus	0.73	0.0497
LSR	Lipolysis Stimulated Lipoprotein Receptor	Cell Membrane	0.65	0.0086
CD36	CD36 Antigen Molecule	Golgi apparatus	0.63	0.0209
CIB1	Calcium- and integrin-binding protein 1	Golgi apparatus	0.62	0.0033
PALM	Paralemmin	Cell Junction	0.62	0.0433
CD109	CD109 Molecule	Cell Membrane	0.59	0.0095
CA4	Carbonic Anhydrase 4	Enzyme	0.58	0.0075
CD9	CD9 Molecule	Extracellular Vesicles	0.57	0.0396
BCAM	Basal Cell Adhesion Molecule	Transmembrane	0.53	0.0337
RAP1B	Ras-related protein Rap-1b	Extracellular Vesicles	0.52	0.0446
EDNRB	Endothelin Receptor Type B	Cell Membrane	0.50	0.00035
**Down - Regulated Proteins**				
**Gene symbol**	**Description**		**Fold change**	**P-value**
PSAP	Prosaposin	Secreted	-0.57	0.0160
PDLIM1	PDZ And LIM Domain 1	Cytoplasm	-0.57	0.0378
SND1	Staphylococcal Nuclease and Tudor Domain Containing 1	Nucleus	-0.62	0.0364
OIT3	Oncoprotein Induced Transcript 3	Nucleus	-0.62	0.0329
CLTB	Clathrin Light Chain B	Extracellular Vesicles	-0.77	0.0139
PDIA3	Protein disulfide-isomerase A3	Endoplasmic Reticulum	-0.78	0.0284
KRT17	Keratin 17	Cytoplasm	-0.79	0.0419
RAD23B	RAD23 Homolog B, Nucleotide Excision Repair Protein	Cytoplasm	-0.82	0.0279
KRT6A	Keratin 6A	Cytoplasm	-0.82	0.0465
TUBA4A	Tubulin Alpha 4a	Cytoplasm	-0.83	0.0380
ACTN1	Actinin Alpha 1	Cell Membrane	-0.85	0.0331
UTRN	Utrophin	Cell Membrane	-0.85	0.0123
CALU	Calumenin	Golgi Apparatus	-0.92	0.0271
S100A2	S100 Calcium Binding Protein A2	Nucleus	-1.14	0.0288
